# Lipophilic versus hydrophilic statin therapy for heart failure: a protocol for an adjusted indirect comparison meta-analysis

**DOI:** 10.1186/2046-4053-2-22

**Published:** 2013-04-23

**Authors:** Kwadwo Osei Bonsu, Amudha Kadirvelu, Daniel Diamond Reidpath

**Affiliations:** 1 School of Medicine and Health Sciences, Monash University Sunway Campus, Jalan Lagoon Selatan, Bandar Sunway, Selangor, DE 46150, Malaysia

**Keywords:** Lipophilic statin, Hydrophilic statin, Statin therapy, Adjusted indirect comparison, Heart failure

## Abstract

**Background:**

Statins are known to reduce cardiovascular morbidity and mortality in primary and secondary prevention studies. Subsequently, a number of nonrandomised studies have shown statins improve clinical outcomes in patients with heart failure (HF). Small randomised controlled trials (RCT) also show improved cardiac function, reduced inflammation and mortality with statins in HF. However, the findings of two large RCTs do not support the evidence provided by previous studies and suggest statins lack beneficial effects in HF. Two meta-analyses have shown statins do not improve survival, whereas two others showed improved cardiac function and reduced inflammation in HF. It appears lipophilic statins produce better survival and other outcome benefits compared to hydrophilic statins. But the two types have not been compared in direct comparison trials in HF.

**Methods/design:**

We will conduct a systematic review and meta-analysis of lipophilic and hydrophilic statin therapy in patients with HF. Our objectives are:

1. To determine the effects of lipophilic statins on (1) mortality, (2) hospitalisation for worsening HF, (3) cardiac function and (4) inflammation.

2. To determine the effects of hydrophilic statins on (1) mortality, (2) hospitalisation for worsening HF, (3) cardiac function and (4) inflammation.

3. To compare the efficacy of lipophilic and hydrophilic statins on HF outcomes with an adjusted indirect comparison meta-analysis.

We will conduct an electronic search of databases for RCTs that evaluate statins in patients with HF. The reference lists of all identified studies will be reviewed. Two independent reviewers will conduct the search. The inclusion criteria include:

1. RCTs comparing statins with placebo or no statin in patients with symptomatic HF.

2. RCTs that employed the intention-to-treat (ITT) principle in data analysis.

3. Symptomatic HF patients of all aetiologies and on standard treatment.

4. Statin of any dose as intervention.

5. Placebo or no statin arm as control.

The exclusion criteria include:

1. RCTs involving cerivastatin in HF patients.

2. RCTs with less than 4 weeks of follow-up.

**Discussion:**

We will perform an adjusted indirect comparison meta-analysis of lipophilic versus hydrophilic statins in patients with HF using placebo or no statin arm as common comparator.

## Background

Statins have been shown to decrease cardiovascular morbidity and mortality in primary [1] and secondary [2] prevention studies. The Cholesterol Treatment Trialists (CTT) collaboration recently reported the findings of prospective meta-analysis of individual data involving about 90,000 patients in 14 randomised trials. Statin therapy was found to reduce the 5-year incidence of major vascular events (defined as coronary death, non-fatal myocardial infarction, coronary revascularisation or stroke) by about one fifth per every 1.0 mmol/l reduction in low-density lipoprotein cholesterol (LDL-C), regardless of the initial lipid profile or other presenting characteristics [3]. The CTT collaboration subsequently reported a further reduction in the incidence of heart attack, coronary revascularisation and ischaemic stroke with an additional 1.0mmol/l reduction of LDL-C. The authors also found that a 2–3 mmol/l reduction of LDL-C would reduce the risk of vascular events by about 40–50% [4]. The most recent meta-analysis of 175,000 patients in 27 randomised trials indicates that in individuals with a 5-year risk of major vascular events lower than 10%, each 1 mmol/l reduction in LDL cholesterol produced an absolute reduction in major vascular events of about 11 per 1,000 over 5 years [5]. These studies clarified the role of statin therapy in the prevention of cardiovascular events and have shown that the benefits of statin therapy exceed any known risks. Nonetheless, it is still unclear whether statins are beneficial in patients with established heart failure. A number of non-randomised studies have shown statin therapy improves clinical outcomes in patients with HF [6-10]. Likewise, small-scale RCTs have shown improved cardiac function and reduced inflammation and mortality outcomes in HF [11-14]. The improved outcomes may be attributed to the cholesterol-lowering effects of the statins, but are more plausibly associated with their pleotropic effects.

In contrast to the non-randomised and small randomised trials, the findings of two recent large RCTs—the Controlled Rosuvastatin Multinational Study in Heart Failure (CORONA) [15] and Gruppo Italiano per lo Studio della Sopravvivenza nell’Insufficienza cardiaca (GISSI-HF) [16]—do not seem to fully support the previous studies. Both trials failed to show significant benefit in primary outcomes compared with placebo. However, the CORONA reported a significant reduction in hospital admissions. It is also reassuring to note that post hoc analyses of CORONA show significantly improved survival in patients with low concentrations of galectin-3 [17] and NT-proBNP [18] from rosuvastatin treatment. Nonetheless, these findings do not recommend the general use of statins in HF but endorse their use in ischaemic heart disease patients with plasma concentrations of galectin-3 and NT-proBNP lower than 19.0 ng/ml and 103 pmol/ml respectively. This observation complements the findings of the Heart Protection Study (HPS) in which patients with low levels of NT-proBNP had improved survival with simvastatin treatment [19]. But the presence of heart failure among study participants was not recorded at baseline, making it impossible to directly estimate the effect of simvastatin in patients with and without heart failure at randomisation. This evidence comes from retrospective analyses, which may thus be considered as hypothesis generating and needs to be confirmed in a prospective study. Furthermore, two meta-analyses of RCTs [20,21] that randomised HF patients to statin or placebo showed no significant improvement in survival. The findings of CORONA and GISSI-HF coupled with those of the two meta-analyses seem to weigh against the use of statin therapy in HF. However, a closer examination of the trials in the meta-analyses suggests that, in combining the data, the researchers did not compare effects of statin types used in the individual studies but treated them as if they were a uniform class of drugs. Within the statins, there are two distinct types identifiable by their lipophilicity. The type of statin used in CORONA and GISSI-HF is believed to have contributed to the unfavourable results in both trials and also may have skewed the outcomes of both meta-analyses towards the results of these two large trials.

Statins are similar in mode of action and comparable in potency to reducing LDL-C; however, they differ in exerting their pleotropic effects. Differences in pleotropic effects are attributable to marked differences in lipophilicity between the types of statins. This occurs because of the presence or absence of polar moieties on their largely hydrophobic structures, which influence solubility and localisation to bring about metabolic differences among the statins [22]. Lipophilic statins enter into cells by passive diffusion and are thus widely distributed in different tissues, whereas hydrophilic statins are liver-specific, employ carrier-mediated mechanisms for uptake and thus could reduce their ability to exert non-lipid effects on extrahepatic tissues [23]. Atorvastatin, simvastatin, lovastatin, fluvastatin, cerivastatin and pitavastatin are lipophilic, while pravastatin and rosuvastatin are hydrophilic. Lipophilic statins appear to exert more beneficial effects in patients with HF, though equivocal outcomes have been reported in a few studies. In contrast, the hydrophilic statins have generally shown more unfavourable effects, though improved outcomes have been reported in a few studies. In particular, hydrophilic rosuvastatin, which is the most potent of all statins in reducing LDL-C, is the statin used in the CORONA and GISSI-HF trials. It is these large trials of a single hydrophilic statin that overwhelm the meta-analyses.

Several studies, subsequent to CORONA and GISSI-HF, have shown that statins may still improve survival and other outcomes in patients with HF [24-26]. These studies, though nonrandomised, again suggest that it is the lipophilic but not hydrophilic statins that may benefit patients with HF [24]. In addition, a meta-analysis that failed to improve overall survival with statin therapy showed in a subgroup analysis that lipophilic statins may improve survival in patients with HF [20]. Interestingly, two other meta-analyses recently showed that statins improve cardiac function [27] and reduce inflammation [28] in patients with HF. Inherent pharmacologic differences among statins may account for observed differences in clinical outcomes of patients. A close observation of these inconsistent and conflicting data shows that the hydrophilicity or lipophilicity of statins accounts for the controversial results obtained in studies in HF. Several prospective and retrospective studies have compared the two types of statins in cardiovascular conditions. The Pravastatin or Atorvastatin Evaluation and Infection Therapy (PROVE-IT) study compared atorvastatin 80 mg with pravastatin 40 mg in a head-to-head fashion in patients with acute coronary syndrome. The PROVE-IT study showed that lipid lowering with lipophilic atorvastatin provides greater protection against death and cardiovascular events than hydrophilic pravastatin although the study did not employ equipotent doses of the two statins [29]. A subanalysis of the Multicenter Study for Aggressive Lipid-lowering Strategy by HMG-CoA Reductase Inhibitors in patients with Acute Myocardial Infarction (MUSASHI-AMI) database found hydrophilic pravastatin to be superior to lipophilic statins in preventing new Q-wave appearance and reducing cardiovascular events in normocholesterolemic patients [30]. However, in a study of patients with coronary artery disease, no significant difference in the incidence of all-cause events was observed with the two types of statins [31]. Furthermore, a more recent study has shown that short-term cardiovascular outcomes were better with lipophilic statins, though long-term outcomes were comparable to those of hydrophilic statins in patients with acute myocardial infarction (AMI). Thus, statin type did not influence long-term outcomes in patients with AMI [32].

However, in HF, little is known about differences in the efficacy of the two types of statins, and it remains unclear which statin type provides better survival and other outcome benefits. Recently, hydrophilic rosuvastatin has been shown to be superior to lipophilic simvastatin in increasing plasma adiponectin and reducing HbA1c levels in patients with non-ischaemic chronic heart failure on standard therapy when compared in a randomised study. Adiponectin is an adipocyte-specific cytokine that has key metabolic effects including insulin sensitivity and predicts cardiovascular events in patients with HF. Haemoglobin A1c (HbA1c) level has been shown to be an independent risk factor for mortality in diabetic and non-diabetic patients with HF [33]. In another study, 63 stable HF out-patients with dilated cardiomyopathy (DCM) on standard therapy were randomised to either atorvastatin (*n =* 32) or rosuvastatin (*n =* 31) and followed up for 6 months. Lipophilic atorvastatin was found to be superior to hydrophilic rosuvastatin in improving cardiac sympathetic nerve activity and reducing plasma NT-proBNP levels in HF patients with DCM [34]. However, these studies were not sufficiently powered to detect significant differences in major outcomes (survival and hospital admissions) in HF. Thus, it remains unclear as to which type of statin—lipophilic or hydrophilic—is more efficacious on outcomes of patients with HF. As yet, there are no adequately powered head-to-head comparison trials to compare the efficacy of the two types of statins in HF. Thus, to compare the efficacy of lipophilic and hydrophilic statins and to investigate which statin subtype provides better survival and other outcome benefits, we will review all available data from RCTs that employed statins in the treatment of HF. We will subsequently conduct an adjusted indirect comparison meta-analysis of the efficacy of lipophilic versus hydrophilic statins in outcomes of HF.

## Methods/Design

### Study objectives

This review seeks to appraise the efficacy of lipophilic and hydrophilic statins on clinical outcomes in heart failure and to compare their efficacy using an adjusted indirect comparison meta-analysis. According to our protocol, the primary outcome is all-cause mortality. We will also examine the effects of statins on cardiovascular mortality, sudden death, hospitalisation for worsening HF, cardiac function and markers of inflammation as secondary outcomes. We will also evaluate the impact of follow-up and statin doses on treatment outcomes in selected studies.

## Inclusion criteria

### Types of studies

Randomised controlled trials comparing statins with placebo or no statin in patients with symptomatic HF will be our main inclusion criterion. Previous meta-analyses of statins in HF will be included and a reference list of selected meta-analyses will be searched. RCTs that employed the intention-to-treat (ITT) principle in the data analysis will be eligible for this review.

### Types of participants

Patients with symptomatic HF, regardless of the aetiology, assigned to statin treatment or control (no statin or placebo) and on standard medical therapy with at least 1 month follow-up will be included in this review.

### Type of interventions

1. Statin at any dose

2. Placebo or no statin as control

### Types of outcome measures

All-cause mortality is an outcome of great importance in HF and will provide the best estimate of treatment effect. The effect of statin treatment on cardiovascular mortality, sudden death, hospitalisation, cardiac function and markers of inflammation will be evaluated.

## Exclusion criteria

Trials involving cerivastatin in patients with HF will be excluded. Cerivastatin has been withdrawn from the market because of the increased incidence of rhabdomyolysis that led to kidney failure among patients who received the full dose (0.8 mg/day) alone or with gemfibrozil [35]. Thus, inclusion of trials involving cerivastatin in HF may introduce bias into the findings of the review. Trials will be excluded if follow-up duration is less than 4 weeks. Trials with crossover designs and post hoc analysis of statins in HF trials will be excluded.

### Search strategy

We will conduct an electronic search of PubMed, MEDLINE, EMBASE, EBM review and Cochrane databases from start dates to January 2013. Basic search terms from combined text and Medical Subject Heading (MeSH) terms including statin, heart failure, randomised controlled trials (#statin AND #heart failure AND #randomised controlled trials) will be used in varying combinations for the search. Table 1 shows the main search strategy for PubMed. This search strategy will be modified to suit each database. The reference list of each identified study will be reviewed for randomised studies. Two independent reviewers will perform the search.

**Table 1:** Search terms and strategy to search PubMed to identify statin studies in heart failure

**Strategy**	**Search terms**
#1	“Right-sided” OR “right sided”
#2	“Left-sided” OR “left sided”
#3	Congestive
#4	Chronic
#5	Diastolic
#6	Systolic
#7	“Heart failure” OR “failure, heart”
#8	#7 AND (#1 OR #2 OR #3 OR #4 OR #5 OR #6)
#9	“Heart decompensation” OR “decompensation, heart”
#10	#8 OR #9
#11	((clinical[Title/Abstract] AND trial[Title/Abstract]) OR clinical trials[MeSH Terms] OR clinical trial[Publication Type] OR random*[Title/Abstract] OR random allocation[MeSH Terms] OR therapeutic use[MeSH Subheading])
#12	“Statin” OR “statins” OR “hydroxymethylglutaryl-CoA reductase inhibitors”
#13	#12 AND #11 AND #10

A search diary will be kept detailing names of the databases searched, keywords used and search results. Titles and abstracts of studies to be considered for retrieval will be recorded in EndNote reference software.

### Selection procedures

**Figure 1:**
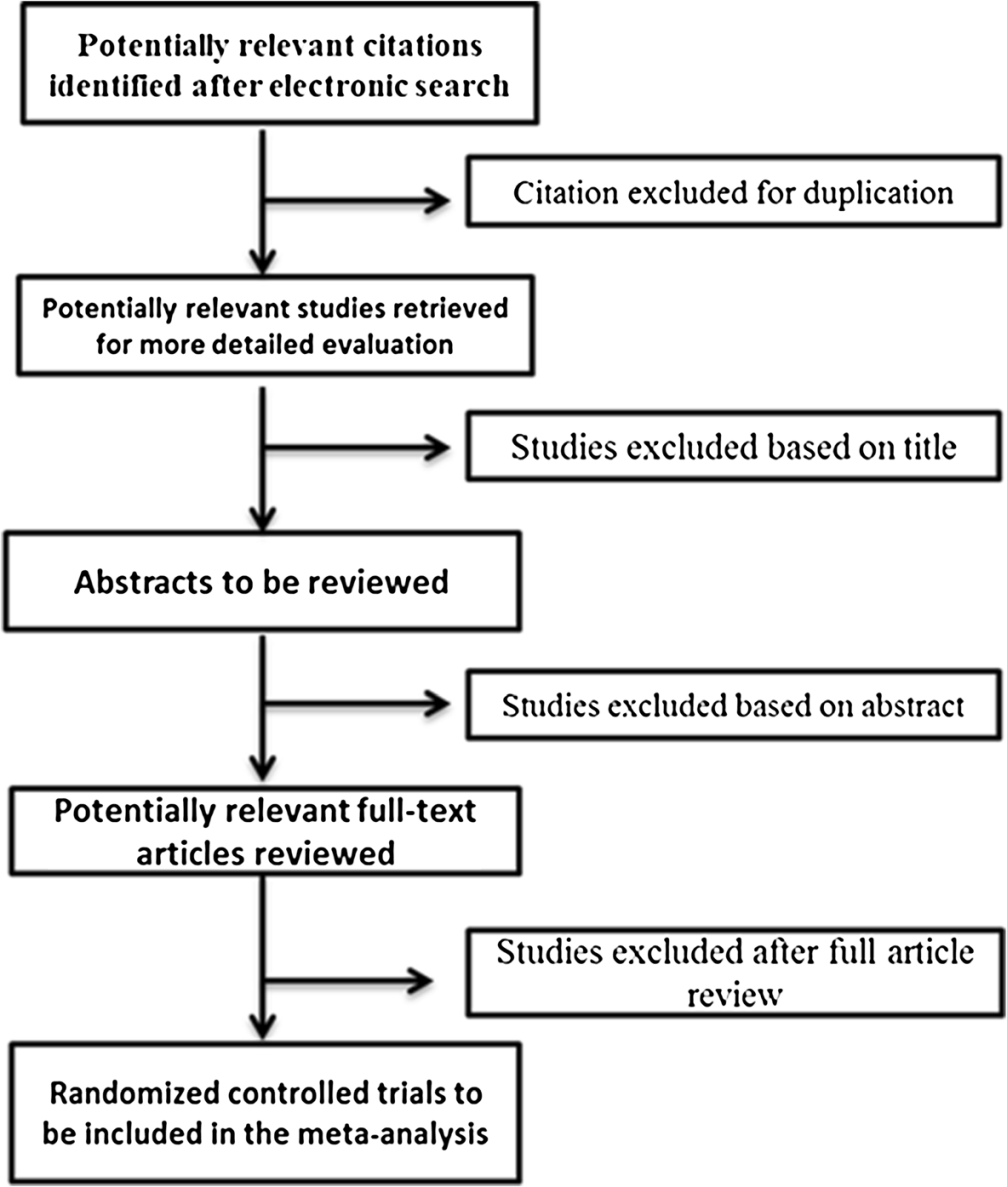
Flow diagram of study selection procedure.

Full-text articles identified by the search and meet the inclusion criteria, based on their title, abstract and subject descriptors will be obtained for data synthesis. Articles identified through reference list searches will also be considered for data collection. Two reviewers will independently select articles according to inclusion criteria. Inconsistencies in selections will be resolved at a meeting between the two reviewers prior to retrieval of selected articles. Retrieved studies will be filed according to inclusion and exclusion decisions. Quality of selected trials for inclusion in the review will be assessed. The Jadad quality scale—a measure of study design that numerically scores studies between 0 and 5 [36] —will be used to evaluate the quality of selected trials. Here the two reviewers will independently assess studies for methodological validity prior to inclusion. Identified studies that meet the inclusion criteria will then be grouped according to the class of statin used in the trial. Figure 1 shows the flow diagram of the study selection procedure.

### Data collection and Statistical analysis

Following assessment of methodical quality, data will be extracted from the identified papers. Data extraction will be done by two reviewers and discrepancies will be resolved by a third reviewer. Data on the number of patients randomised to active treatment, placebo or control, type of statin, dose, loss to follow-up, level of blinding, concealment of allocation, specific study inclusion and exclusion criteria, duration of follow-up, hospitalisation and deaths will be abstracted from each study. Authors of selected studies will be contacted for clarification when necessary.

Dichotomous outcome variables will be computed as odd ratios (OR) with 95% confidence intervals (CIs) using Peto’s one-step method for fixed-effects model [37]. From previous meta-analysis, it is anticipated that most of the eligible studies to be selected for this review will have small sample sizes and may have no or similar events in the control and experimental arms of studies. Thus, the choice of Peto’s one-step approach will be the most appropriate method for our meta-analysis. For each study, the “observed minus expected” statistic (O - E) and its variance (v) will be calculated from the number of participants that had the dichotomous outcomes and the total number of participants in each treatment arm. These (O - E) values, one from every study, will be summed to produce a grand total (Gt), with its variance (V) equal to the sum of their separate variances. The value exp (Gt/V) is Peto’s one-step estimate of the odds ratio, and its 95% confidence interval will be exp (Gt/V ± 1.96/√V). For continuous outcomes, endpoints will be based on the change from baseline to follow-up. Pooled effects of continuous outcome variables will be presented as standardised mean differences (SMD) with 95% CIs using the inverse variance approach for the fixed-effects model. We anticipate systematic differences between the results of studies (heterogeneity); therefore, pooled effects estimates for both continuous and dichotomous outcomes will also be computed with the random-effects approach of DerSimonian & Laird [38].

In the absence of randomised trials sufficiently powered to make head-to-head comparisons of lipophilic to hydrophilic statins, an indirect comparison is plausible using a common comparator (for this study, placebo or no statin arm) [39-41]. Meta-analysis of trials comparing lipophilic statins to control (placebo or no statin) will be conducted to obtain an estimate of treatment effect. This will be preceded by a separate meta-analysis of trials comparing hydrophilic statins to control. Statistical heterogeneity of all pooled outcome measures in both meta-analyses will be assessed with the I squared (I^2^) statistics (I^2^ > 50%; treatment effect will be considered as statistically inconsistent) [42]. Meta-regression and subgroup analysis will be performed to explore heterogeneity among selected trials. The principle of meta-regression and subgroup analysis will be employed to evaluate the impact of several moderators including patient age, sex, HF aetiology, severity of HF, different statin doses, baseline LDL cholesterol and study follow-up duration on treatment outcomes of selected studies. The various statin doses employed in the selected trials will be categorised into standard and intensive dosage regimens. A meta-regression model will be used to assess the impact of these moderators on treatment outcomes in HF. We will perform sensitivity test to determine the robustness of the pooled estimates by exclusion of one study at a time from the analysis. Publication bias and small study effects will be assessed with the Begg’s adjusted-rank correlation [43] and Egger’s regression asymmetry tests [44]. To conduct an adjusted indirect comparison meta-analysis, the estimated difference in treatment effect of lipophilic and hydrophilic statins in HF will be determined by comparing the two estimated treatment effects using the Bucher’s approach [39]. All analyses will be performed in the R statistical environment [45] and the Indirect Meta-analysis Tool (METCARDIO, Turin, Italy).

## Discussion

This study aims to provide evidence to show whether lipophilic statins are superior to hydrophilic statins on clinical outcomes of heart failure. The lack of direct evidence from head-to-head comparison of the two types of statins in patients with HF has necessitated our execution of an adjusted indirect comparison meta-analysis. Unlike other methods, an adjusted indirect comparison employs a common comparator, which for this study is the placebo or no statin arm of selected trials. This method ensures that the within-trial randomisation is maintained and preserves certain strengths of randomised allocation of patients for estimating comparative effects of treatment [39,46], thus minimising biases. In addition, results of indirect comparison of competing interventions appear to be consistent with those of direct head-to-head comparison trials [41,47]. To the best of our knowledge, this is the first indirect meta-analysis comparing the efficacy of lipophilic to hydrophilic statins on clinical outcomes in patients with HF to be performed. Although indirect comparisons cannot replace evidence from direct comparison trials, it could inform clinicians on choice of treatment among competing interventions such as lipophilic and hydrophilic statins in HF. Until randomised trials directly comparing the two types of statins become available, indirect comparison may provide useful information to guide clinical decision-making. At this time, we are unaware of any planned studies to undertake such direct comparisons. Thus, we will perform an adjusted indirect comparison of lipophilic versus hydrophilic statins using placebo or no statin arm as common comparator.

## Competing interests

The authors declare that they have no competing interests.

## Authors’ contributions

KOB and AK contributed to the conception and design of the review. KOB and DDR will analyse the data and all three authors will participate in the interpretation of the results. All authors were involved in the drafting of this protocol and have given their approval for publication.
